# Pregnancy induced displacement of preexisting microchimeric cells in the absence of maternal B and T cells

**DOI:** 10.3389/fimmu.2024.1478465

**Published:** 2024-10-30

**Authors:** Giang Pham, Tzu-Yu Shao, Jeremy M. Kinder, Yanyan Peng, Lucien H. Turner, Sing Sing Way

**Affiliations:** Division of Infectious Diseases, Center for Inflammation and Tolerance, Cincinnati Children’s Hospital Medical Center, Department of Pediatrics, University of Cincinnati College of Medicine, Cincinnati, OH, United States

**Keywords:** microchimerism, RAG1, T cell, B cell, non-inherited maternal antigen, microchiome, immune tolerance, reproductive fitness

## Abstract

Bidirectional exchange of cells between mother and fetus occurs during pregnancy, and persistence of these genetically foreign cells establishes long-term microchimerism in both individuals after parturition. Since women can have multiple pregnancies, and all mothers were once daughters themselves, the microchimeric milieu in each woman could theoretically contain cells from a variety of origins, including from their own mothers as well as their babies from each pregnancy. Interestingly and in sharp contrast to this prediction, we recently showed preexisting populations of microchimeric cells are lost following pregnancy and associated with seeding of new fetal microchimeric cells. Complete loss of preexisting microchimeric cells in this context draws parallels to immunological rejection with synchronized elimination of cells and tissues that express defined discordant antigens. This perspective evaluates this provocative hypothesis regarding pregnancy induced rejection of microchimeric cells, including new experimental data comparing microchimerism levels in mice simultaneously lacking B and T cells before pregnancy, and after parturition with primary and secondary pregnancies.

## Introduction

Presence of genetically discordant cells in individuals is called chimerism, and persistence of these cells at very low frequency (<1 in 10^5^ cells) is called microchimerism (1–6). Although chimerism is intentionally induced after solid-organ and stem cell transplantation, microchimerism primarily stems from the bidirectional exchange of cells between mother and fetus during pregnancy, with persistence of these cells in both individuals after parturition. Persistence of fetal cells in mothers is called fetal microchimerism, and includes classical examples of Y chromosome expressing cells in the peripheral blood of mothers decades after giving birth to sons (7). Reciprocally, persistence of maternal cells in offspring after delivery is called maternal microchimerism, classically demonstrated by cells expressing two X chromosomes in male offspring (8).

Directly related to the presence of maternal microchimeric cells is expanded immune tolerance in offspring that extends to non-inherited maternal antigen (NIMA) that these cells express (9, 10). Classical examples include resistance to Rhesus (Rh) sensitization in Rh negative women born to Rh positive mothers (11), selective B cell hypo-responsiveness to noninherited maternal HLA haplotypes in transfusion dependent individuals (12), and prolonged renal allograft survival in sibling donor-recipient pairings when matched for NIMA compared with noninherited paternal MHC haplotypes (13). To further dissect the immunological basis responsible for NIMA-specific tolerance, we previously investigated the “chicken and egg” relationship between expanded tolerance in this context and persistence of NIMA-expressing maternal microchimeric cells. We showed experimental depletion of maternal microchimeric cells overturned NIMA-specific tolerance, thereby solidifying their necessity for sustained tolerance in offspring (14). In particular, allogeneic pregnancies sired by males expressing NIMA-matched MHC haplotypes associated with expanded accumulation of immune-suppressive FOXP3+ regulatory T cells (Tregs) with NIMA-specificity were highly resilient to fetal wastage induced by perturbations in fetal tolerance (14). These phenotypes, including protection against fetal wastage and NIMA-specific Treg accumulation, were each overturned in mice depleted of maternal microchimeric cells (14). Given the dominant role reproductive fitness plays in trait selection, cross-generational reproductive fitness driven by maternal microchimeric cells in this context highlights one aspect of nature’s intent with what seems like added immunological complexity associated with individuals being constitutively chimeric (1).

Building upon this line of reasoning that maternal microchimeric cells promote reproductive fitness by enforcing fetal tolerance in the future pregnancies of their daughters, we reasoned partner specific protection conferred by prior pregnancy against complications in future pregnancy might similarly require fetal microchimeric cells that persist in mothers after parturition (15–17). Considering all mothers were each at one time daughters themselves, this reasoning further led to the expectation of an active “microchiome” comprised by an assortment of antigenically diverse genetically foreign cells purposefully retained in reproductive age females to promote reproductive fitness (18). Besides for reproductive benefits, microchimeric cells may at the same time confer other physiological benefits related to tissue regeneration and repair (19–22), but also with potential offsetting negative proinflammatory consequences related to autoimmune disorders such as type 1 diabetes and scleroderma (23–25). Together, these considerations opened up new questions regarding how the assortment of microchimeric cells from unique genetic origin interact with each other in mothers, and whether the niche for microchimeric cells is fixed or capable of expansion in perpetuity.

These questions were recently addressed by evaluating how pregnancy with seeding of new fetal microchimeric cells impacts levels of maternal microchimeric cells; and how secondary pregnancy impacts levels of fetal microchimeric cells retained in mothers from prior pregnancy. In each of these contexts, pregnancy with seeding of new fetal microchimeric cells was shown to displace preexisting microchimeric cells in maternal tissues (26). Preexisting maternal microchimeric cells retained in female mice were eliminated to near completion and replaced by fetal microchimeric cells retained in parous microchimeric cells after primary pregnancy. Likewise, fetal microchimeric cells retained in parous female mice after primary pregnancy were eliminated to near completion and replaced by fetal microchimeric cells from the second pregnancy. Loss of preexisting maternal and fetal microchimerism was surprising given how rare maternal and fetal microchimerism exists in maternal tissues each on the order of 1 in 10^5^ to 10^7^ cells (26). Why can't tissues contain another set of cells of such rarity – similar to how the compartment of memory T cells grows in size with immunological experience (27)?

Another consideration relates to the efficiency whereby preexisting microchimeric cells are eliminated after pregnancy. Efficient loss of preexisting microchimeric cells in this context draws parallels to immunological rejection with synchronized elimination of cells which express defined discordant antigens. These considerations led us to refocus our established platform for measuring maternal and fetal microchimerism to investigate whether similar dynamics and replacement occurs in mothers simultaneously lacking B and T cells, and to evaluate whether these adaptive immune components driving classical rejection phenotypes are essential for pregnancy induced displacement of preexisting maternal and fetal microchimeric cells.

## Maternal B and T cells non-essential for fetal microchimeric cell displacement

**Figure 1:**
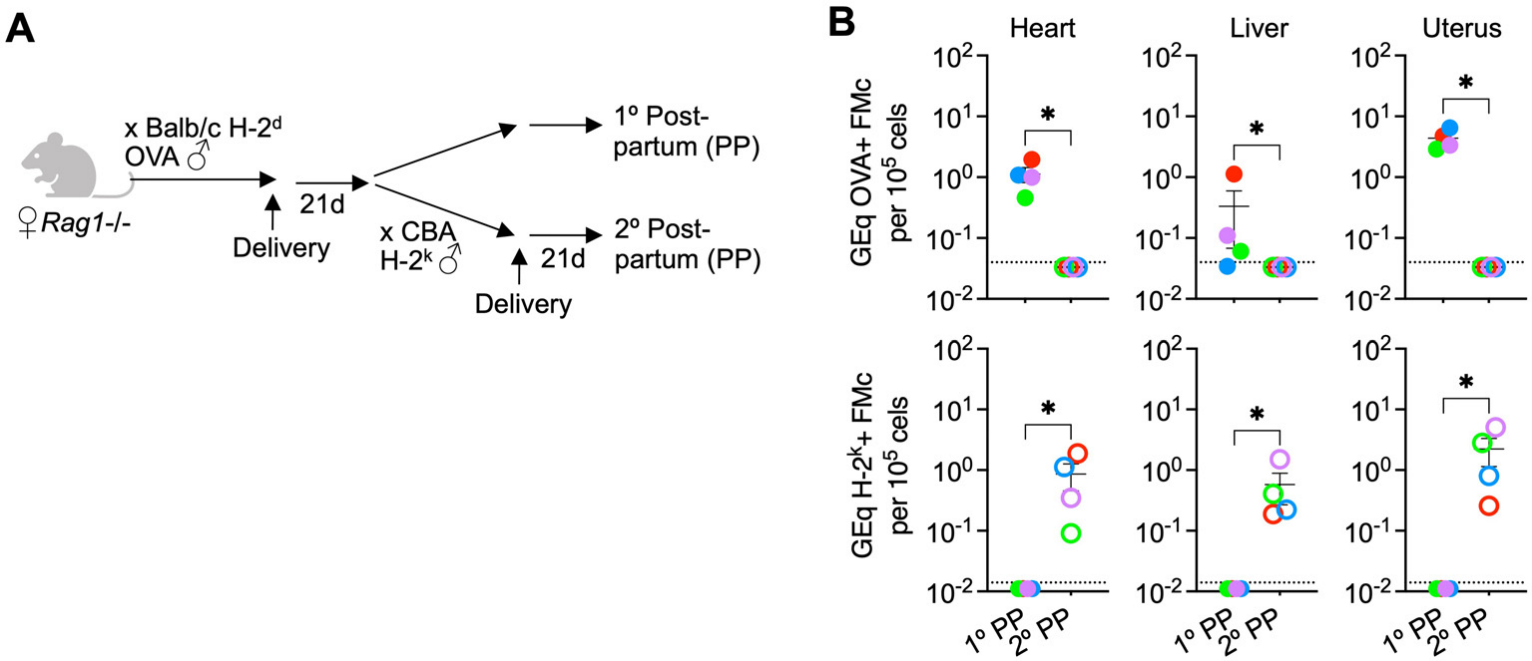
Fetal microchimeric cells displaced after pregnancy despite lack of maternal B and T cells. **(A)** Mating scheme for *Rag1*-/- (Jackson laboratory, strain #002216) sired by OVA+ transgenic males allowing identification of microchimeric cells by quantifying OVA+ DNA (primary postpartum) or after secondary pregnancy sired by non-transgenic CBA H-2^k^ males allowing identification of microchimeric cells by quantifying H-2^k^+ DNA (secondary postpartum). **(B)** Levels of OVA+ DNA specific to OVA+ fetal microchimeric cells or H-2^k^+ DNA specific to H-2^k^+ fetal microchimeric cells in each tissue among *Rag1*-/- mice 21 days postpartum after primary allogeneic pregnancy sired by OVA+ H-2^d^ Balb/c males (filled), or secondary pregnancy sired by H-2^k^+ CBA males (open) quantified using DNA extraction and quantitative PCR methods described in reference (26). Each point represents the data from an individual mouse, with colors used to depict data from tissues of the same mouse, and representative of at least two independent experiments each with similar results. Bar, mean ± standard error. *P < 0.05. Mouse figures were prepared using Biorender; graphs and statistics analyzed using Graphpad Prism (Version 10.1).

For investigating the necessity of maternal B and T cells, *Rag1*-/- female mice deficient in these adaptive immune components on the H-2^b^ C57BL/6 background were used for breeding (28). Similar to our recently published breeding scheme, primary allogeneic pregnancy were sired by transgenic male mice ubiquitously expressing a recombinant cell surface protein containing ovalbumin (OVA) in all cells on the H-2^d^ Balb/c background so that fetal microchimeric cells retained after primary pregnancy can be enumerated by quantifying OVA+ DNA levels (**Figure 1A**) (14, 26). Thereafter for a subset of mice, non-transgenic (non-OVA expressing) H-2^k^+ third party male mice on the CBA background were used to sire secondary pregnancy so that H-2^k^+ fetal microchimeric cells seeded in secondary pregnancy can be distinguished from OVA+ fetal microchimeric cells seeded after primary pregnancy (**Figure 1A**).

These experiments showed consistent recovery of OVA+ fetal microchimeric cells after primary pregnancy in the tissues of *Rag1*-/- female mice (**Figure 1B**), and to levels similar to that previously described for B and T cell sufficient WT mice (26). Remarkably, and in contrast to the prediction that rejection by maternal adaptive immune cells drives displacement of these fetal microchimeric cells, OVA+ fetal microchimeric cells declined to below the limits of detection (1 in 3.3 x 10^6^ cells) in the heart, liver and uterus of *Rag1*-/- female mice after secondary pregnancy sired by H-2^k^+ CBA males (**Figure 1B**). For evaluating potential microchimeric cell displacement, we focused particularly on the heart, liver and uterus because these tissues consistently contain the highest levels of microchimeric cells (14, 26). Loss of OVA+ fetal microchimeric cells seeded in primary pregnancy was associated with the accumulation of H-2^k^+ microchimeric cells after secondary pregnancy compared with H-2^k^+ cells consistently below the limits of detection (1 in 10^7^ cells) in the tissues of *Rag1*-/- control mice after primary pregnancy but without secondary pregnancy (**Figure 1B**). Our interpretation of these results is that maternal B and T cells are simultaneously non-essential for pregnancy induced displacement of fetal microchimeric cells, given similar levels of OVA+ fetal microchimerism after primary pregnancy, and replacement by H-2^k^+ fetal microchimeric cells after secondary pregnancy in *Rag1*-/- female mice.

## Maternal B and T cells non-essential for maternal microchimeric cell displacement

**Figure 2:**
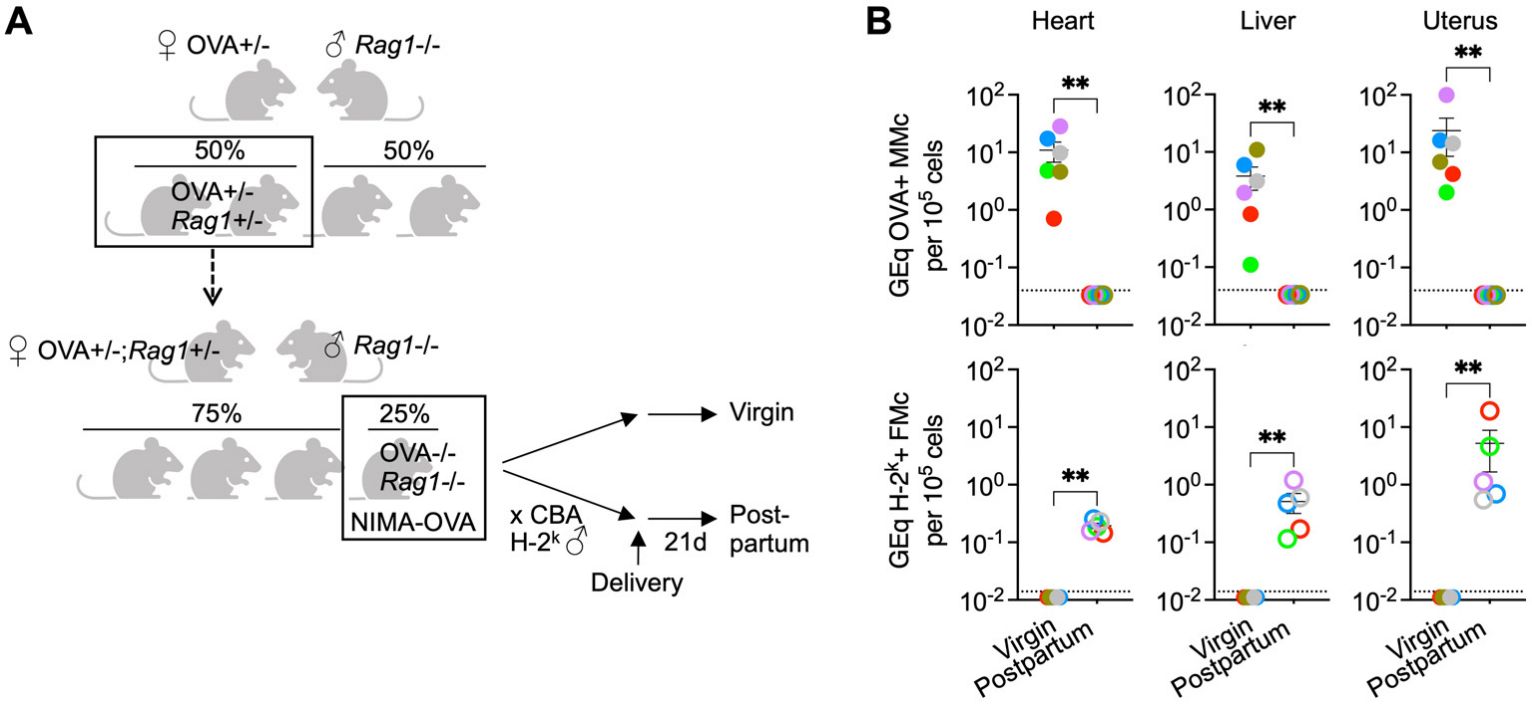
Maternal microchimeric cells displaced after pregnancy despite lack of maternal B and T cells. **(A)** Mating scheme for generating *Rag1*-/- born to OVA+/- mothers transforming OVA into a surrogate NIMA and tracking maternal microchimeric cells after quantifying OVA+ DNA in virgin control or after pregnancy sired by non-transgenic CBA H-2^k^ males (postpartum). **(B)** Levels of OVA+ DNA specific to OVA+ maternal microchimeric cells or H-2^k^+ DNA specific to H-2^k^+ fetal microchimeric cells in each tissue among *Rag1*-/- NIMA-OVA mice 21 days postpartum after allogeneic pregnancy sired by H-2^k^+ CBA males (open) or age-matched virgin *Rag1*-/- NIMA-OVA mice (filled) quantified using DNA extraction and quantitative PCR methods described in reference (26). Each point represents the data from an individual mouse, with colors used to depict data from tissues of the same mouse, and representative of at least two independent experiments each with similar results. Bar, mean ± standard error. **P < 0.01. Mouse figures were prepared using Biorender; graphs and statistics analyzed using Graphpad Prism (Version 10.1).

We next evaluated the necessity for maternal B and T cells for pregnancy induced displacement of maternal microchimerism by comparing levels of maternal microchimeric cells in *Rag1*-/- female mice after primary pregnancy compared with *Rag1*-/- virgin control mice. Using our validated breeding scheme (14, 26), non-transgenic female *Rag1*-/- offspring born to *Rag1*+/- mothers heterozygous for the aforementioned transgene encoding constitutive expression of a recombinant cell surface protein containing OVA were utilized, allowing maternal microchimeric cells retained in offspring to be enumerated by quantifying OVA+ DNA levels (**Figure 2A**). Thereafter for investigating how fetal microchimeric cells seeded in pregnancy impacts levels of these maternal microchimeric cells, non-transgenic H-2^k^+ third party male mice on the CBA background were used to sire primary pregnancy so that H-2^k^+ fetal microchimeric cells can be distinguished from preexisting OVA+ maternal microchimeric cells (**Figure 2A**).

These experiments showed the expected recovery of OVA+ maternal microchimeric cells in the heart, liver and uterus of *Rag1*-/- virgin female offspring (**Figure 2B**), and to levels similar to that previously described for B and T cell sufficient WT mice (26). Similar to non-essential roles for maternal B and T cells for displacement of fetal microchimerism, OVA+ maternal microchimeric cells consistently declined to below the limits of detection (1 in 3.3 x 10^6^ cells) in maternal tissues of *Rag1*-/- female mice after primary pregnancy sired by H-2^k^+ CBA males (**Figure 2B**). Loss of OVA+ maternal microchimerism was associated with the accumulation of H-2^k^+ fetal microchimeric cells after primary pregnancy, compared with H-2^k^+ cells consistently below the limits of detection (1 in 10^7^ cells) in the tissues of virgin control *Rag1*-/- mice (**Figure 2B**). Our interpretation of these results is that maternal B and T cells are simultaneously non-essential for pregnancy induced displacement of maternal microchimeric cells given similar OVA+ maternal microchimerism levels in *Rag1*-/- virgin mice, and their efficient replacement by H-2^k^+ fetal microchimeric cells after primary pregnancy in *Rag1*-/- female mice.

## Fetal B and T cells non-essential for microchimeric cell displacement

Although using non-leaky *Rag1*-/- mice conclusively demonstrates non-essential roles for maternal B and T cells, we recognize that microchimerism may also extend to these adaptive immune cells. For example, preconceptually primed maternal pathogen-specific T cells have been described to be vertically transferred and protect neonatal mice against infection (29), and microchimerism in fetal or maternal antibody producing cells are associated with accumulation of arthritis-specific autoantibodies (30). Thus to further investigate the necessity of newly seeded fetal microchimeric B and T cells for displacement of preexisting microchimeric cells, *Rag1*-/- males on the C57BL/6 background were used instead of CBA H-2^k^ males in the aforementioned platforms for evaluating microchimeric cell displacement.

We reasoned use of *Rag1*-/- male to sire secondary pregnancy in *Rag1*-/- females with primary pregnancy sired by OVA expressing H-2^d^ Balb/c allows the importance of fetal microchimeric B and T cells in displacing OVA+ fetal microchimeric cells to be evaluated (**Figure 1A**). Likewise, use of *Rag1*-/- male to sire primary pregnancy in non-transgenic female *Rag1*-/- offspring born to *Rag1*+/- mothers heterozygous for the OVA transgene allows evaluating the necessity for fetal microchimeric B and T cells in displacing OVA+ maternal microchimeric cells (**Figure 2A**). Together these experiments showed non-essential roles for fetal microchimeric B and T cells in displacement of preexisting microchimeric cells. OVA+ DNA was reduced to below the limits of detection (1 in 3.3 X 10^6^ cells) in the heart, liver and uterus for *Rag1*-/- females with primary pregnancy sired by OVA expressing H-2^d^ Balb/c mice after secondary pregnancy with *Rag1*-/- males (6 of 6 tissues from 2 postpartum mice). In turn, OVA+ DNA was similarly reduced to below the limits of detection in the heart, liver and uterus for *Rag1*-/- virgin females with OVA+ maternal microchimeric cells after primary pregnancy sired by *Rag1*-/- males (12 of 12 tissues from 4 postpartum mice). Along with demonstrating non-essential roles for fetal microchimeric B and T cells in loss of preexisting microchimeric cells, use of *Rag1*-/- males on the C57BL/6 H-2^b^ background for establishing syngeneic pregnancy also show displacement in this context is not limited only allogeneic pregnancy.

## Discussion

One of the most fascinating and perplexing immunological considerations pertaining to microchimeric cells is how these cells persist and escape rejection, despite being genetically foreign. Persistence in this context appears to be life-long, given recovery of fetal microchimeric cells in women decades after giving birth and recovery of maternal microchimeric cells in adult individuals (1, 2, 5, 7). An interesting new twist to this conundrum is how pregnancy appears to so efficiently reset the microchimeric cell niche, with near complete replacement of preexisting microchimeric cells with new fetal microchimeric cells. Herein we describe our rationale and experience investigating the hypothesis that loss of preexisting microchimeric cells reflects rejection by evaluating this process using Rag1-deficient mice simultaneously lacking B and T cells. Remarkably, we find absence of these adaptive immune components known to drive rejection of genetically foreign cells and tissues in transplantation does not override pregnancy induced displacement of either preexisting maternal microchimeric cells in mice after primary pregnancy, or preexisting fetal microchimeric cells seeded by primary pregnancy in mice after secondary pregnancy. We recognize that adaptive features associated with rejection have been described for more traditional innate immune cell types including natural killer cells, innate lymphoid cells and monocyte-macrophage cells remaining in *Rag1*-/- mice. For example, natural killer cells are capable of context-specific self versus non-self discrimination, and with transplant induced activation licensed to kill allogeneic target cells (31). Phenotypically distinct proinflammatory compared with protective innate lymphoid cells are increasing associated with allograft rejection versus acceptance (32, 33), and macrophage cells with trained activation may reduce the threshold of stimulation T cell mediated allograft rejection (34). Thus evaluating the contribution of these more classical innate immune cells in pregnancy induced microchimerism dynamics and displacement, including earlier time points after parturition, are important areas for future investigation.

Perhaps a broader consideration is whether viewing loss and replacement of microchimeric cells through the lens of traditional rejection and loss of tolerance to non-self antigens expressed by microchimeric cells is too simplistic. For example, second pregnancy induced loss of fetal microchimeric cells seeded in primary pregnancy does not functionally erase tolerance to fetal antigens encountered in first pregnancy. We recently showed enforced tolerance is sustained after encounter with these same antigens in tertiary pregnancy through expanded accumulation of FOXP3+ Tregs derived from a pool of cells that lose FOXP3-expression called exTregs (26). Thus, if rejection drives the loss of preexisting fetal microchimeric cells allowing seeding of new fetal microchimeric cells in subsequent pregnancy, it is difficult to imagine why tolerance is not overturned, and instead reinforced. This example also illustrates an interesting distinction between how fetal microchimeric cells and maternal microchimeric cells prime tolerance. Tolerance to NIMA appears to be completely erased after depletion or displacement of maternal microchimerism, whereas tolerance to fetal antigens encountered even in past pregnancies seem to be retained in mothers despite intervening pregnancy sired by genetically discordant male partners (26, 35). Thus, outstanding questions remain regarding whether NIMA-specific tolerance is transformed to NIMA-specific sensitization in women after pregnancy, and especially with male partners without overlapping NIMAs. Here a tantalizing consideration is the biphasic effects on renal allograft survival where NIMA-matching leads to a higher incidence of early rejection in some individuals despite overall improved long-term allograft survival (13). If rejection drives loss of maternal microchimeric cells, we would hypothesize parous women would be highly enriched among individuals with early rejection, as a complementary explanation to “split tolerance” for the biphasic response to NIMA-matched tissues (36).

Regardless of whether classical rejection mechanisms are applicable for microchimeric cell replacement, another fascinating consideration is the surprisingly fixed niche of these exceptionally rare cells that appears to allow the presence of cells from only a single source at a time. Data we present here further show non-essential roles for B and T cells in restricting this remarkably fixed niche given efficient replacement of maternal microchimeric cells by fetal microchimeric cells in *Rag1*-/- after primary pregnancy, and similarly efficient replacement of preexisting fetal microchimeric cells in *Rag1*-/- mothers by new fetal microchimeric cells in subsequent pregnancy. How this physiological niche is controlled, and apparently reset by pregnancy remains uncertain. Given the wide heterogeneity in maternal microchimeric and fetal microchimeric cell levels within each tissue and across multiple tissues in individual mice (14, 26), this niche is unlikely to be simply numerical where individual tissues can only accommodate a fixed number of rare microchimeric cells. If not numerical and not immunological, how does it work?

One intriguing hypothesis for why microchimerism may be restricted to cells from only a single source at a time is to minimize immunological complexity. By extension, inefficient or incomplete microchimeric cell replacement allowing for multiple sets of genetically discordant cells to be present in women at the same time leading to more immunological complexity may increase the risk of autoimmune or autoinflammatory disorders. Thus, incomplete microchimeric cells replacement may offer an additional explanation for increased incidence of autoimmune disorders in women compared with men (37–39). Potential negative consequences of microchimerism in this context is consistent with the average age of onset after puberty for most autoimmune disorders that disproportionately impact women including systemic lupus erythematosus, systemic sclerosis, and rheumatoid arthritis (40). Comparatively, since males with maternal microchimeric cells do not become pregnant, displacement by fetal microchimeric cells should never occur, precluding expanded immunological complexity caused by inefficient or incomplete microchimeric cell replacement. Thus, additional important areas for future investigation include evaluating levels of microchimeric cells from a variety of likely sources (maternal microchimerism; fetal microchimerism from each pregnancy) in the circulation and diseased tissues of parous women with autoimmunity.

In the larger scientific context, while pregnancy has been and remains arguably the most physiological model of immune tolerance, it remains underutilized for investigating how immune cells work. Systemic seeding of fetal cells into the tissues of women during pregnancy may represent only a small part of the larger puzzle for why mothers do not reject their babies during pregnancy, why babies do not reject their mothers, and pregnancy induced shifts in severity of systemic and organ-specific autoimmune disorders. Within this reproductive framework and behind the scenes, there also appears to be dynamic forces allowing for near complete displacement of preexisting microchimeric cells and replacement by new fetal microchimeric cells in maternal tissues during pregnancy. We are just beginning to scratch the surface in unraveling how our bodies work and reproduce, including the importance of these exceptionally rare but intriguing cells that defy classical immunological rules related to tolerance and rejection. These points are further highlighted in this brief perspective suggesting similar non-classical means for the displacement and replacement of these cells which occurs with remarkable efficiency. In turn understanding what microchimeric cells do and how they work, including synchronized disappearance to make space for a new set of cells during pregnancy, holds exceptional promise for innovative new ways for investigating how to promote acceptance or rejection of genetically foreign cells in other physiological contexts including transplantation and cancer.

## Data Availability

The original contributions presented in the study are included in the article/supplementary material. Further inquiries can be directed to the corresponding author.
